# Prokaryotic virus host prediction with graph contrastive augmentaion

**DOI:** 10.1371/journal.pcbi.1011671

**Published:** 2023-12-01

**Authors:** Zhi-Hua Du, Jun-Peng Zhong, Yun Liu, Jian-Qiang Li

**Affiliations:** College of Computer Science and Software Engineering, Shenzhen University, Shenzhen, Guang-dong, China; Emory University Department of Biology, UNITED STATES

## Abstract

Prokaryotic viruses, also known as bacteriophages, play crucial roles in regulating microbial communities and have the potential for phage therapy applications. Accurate prediction of phage-host interactions is essential for understanding the dynamics of these viruses and their impacts on bacterial populations. Numerous computational methods have been developed to tackle this challenging task. However, most existing prediction models can be constrained due to the substantial number of unknown interactions in comparison to the constrained diversity of available training data. To solve the problem, we introduce a model for **p**rokaryotic virus **h**ost **p**rediction with **g**raph **c**ontrastive **a**ugmentation (PHPGCA). Specifically, we construct a comprehensive heterogeneous graph by integrating virus-virus protein similarity and virus-host DNA sequence similarity information. As the backbone encoder for learning node representations in the virus-prokaryote graph, we employ LGCN, a state-of-the-art graph embedding technique. Additionally, we apply graph contrastive learning to augment the node representations without the need for additional labels. We further conducted two case studies aimed at predicting the host range of multi-species phages, helping to understand the phage ecology and evolution.

## Author summary

Prokaryotic viruses, which specifically target and infect bacteria, are highly prevalent in various ecosystems, establishing a dynamic relationship with their bacterial hosts. As the most abundant biological entities on Earth, phages are present wherever bacteria coexist. Therefore, accurately predicting phage-host interactions is of great significance. However, most existing prediction approaches primarily focus on learning sequence-based features for host prediction, disregarding the valuable information inherent in virus-virus and virus-prokaryote interactions. In this study, we propose a novel method, enabling accurate prediction of phage hosts. The model surpasses state-of-the-art methods in terms of host prediction performance across three benchmark datasets. Furthermore, it demonstrates the capability to predict the host range of multi-species phages, thus facilitating their practical application in various domains.

## Introduction

Prokaryotic viruses, including phages and archaeal viruses, play a crucial role in diverse ecosystems such as limnetic, marine, and soil systems [1–4]. Viruses represent the most extensive group of organisms harboring unexplored genetic diversity. The application of metagenomic techniques has facilitated a rapid surge in the identification of novel viruses. Among them, phages, which are integral components of the human microbiota, have been shown to exert influence on gut health and the development of certain diseases. [5] Furthermore, the escalating challenge of combating antibiotic resistance in bacteria poses a serious threat to the effective control of bacterial infectious diseases [6], phages are also used as therapy for treating diseases caused by bacteria [7]. As viruses are unable to survive independently, investigating their host is crucial. Prokaryotic viruses typically have specific hosts and inject their genetic material into host cells, utilizing low-molecular substances to propagate. Despite the recognition among researchers of the significance of the interaction between prokaryotic viruses and hosts, traditional laboratory culture experiments are time-consuming and expensive [8]. More critically, less than 1% of microbial hosts have been cultivated in the laboratory [9, 10]. Hence, there is a pressing need to develop computational tools for accurately identifying prokaryotic virus hosts. In recent times, various computational approaches have been employed for host prediction, which can be broadly categorized into two groups: alignment-based methods and alignment-free methods. Alignment-based methods rely on sequence similarity between viruses and prokaryotes, as gene fragments may be shared between them. Such gene fragment sequences are from the spacer sequences for the CRIPSR [11] system. These sequences are obtained from DNA fragments of viruses that have previously infected the prokaryotes. When these viruses with recorded sequences attack again, the prokaryotes can employ CRISPR-based mechanisms to destroy the viral DNA and protect themselves. Thus, CRISPR can be considered as compelling evidence of virus-prokaryote interactions, owing to its infection and protection mechanism. However, the use of CRISPR-based evidence to identify interactions between viruses and prokaryotes is limited, as only 40%-70% of prokaryotes encode a CRISPR system [11], and many lack spacer sequences from viruses. BLAST [12] is another widely used alignment-based method for predicting the host of viruses. It identifies short, similar segments between the query and database sequences, and provides information on similarities and differences between the two sequences. Compared to the CRISPR-based approach, the BLAST-based approach generally exhibits lower accuracy but can be applied to a wider range of viruses. However, even though some sequences may have exact matches, they may fail to provide information for host prediction, such as conserved sequences around integration sites [13, 14].

Alignment-free approaches are more flexible as they do not rely on direct sequence comparison. One commonly used approach is the utilization of k-mers, which are short subsequences of fixed length (k) that can be used to identify similar sequences or characterize the composition of a new sequence [15]. K-mer-based methods are computationally efficient and can be applied to a wide range of sequence data, including viruses and prokaryotes, without the need for sequence alignment. They are particularly useful for identifying similarities and patterns in large datasets and can be utilized in host prediction algorithms as a feature extraction technique. VirHostMatcher (VHM) [16], Phage-Host Interaction Search Tool (PHIST) [17] and prokaryotic virus host predictor (PHP) [18] are based on k-mer features to predict the virus hosts. VHM predicts the putative host of each input virus by leveraging similarity in oligonucleotide frequency patterns between the virus and potential hosts, and selecting the one with the smallest dissimilarity. On the other hand, PHIST predicts prokaryotic hosts of viruses by identifying exact matches between viral and host genomes using the Kmer-db tool and PHP employs k-mer features to train a Gaussian mixture model for host prediction. Deep learning-based approaches integrate the information of sequences and artificial neural networks like convolutional neural networks (CNNs) [19] to make predictions. For example, DeepHost [20] applies CNN architecture and treats the host prediction task as a multi-classification problem. The VHM-net [21] constructs a network containing heterogeneous features between viruses and prokaryotes and uses the Markov random field to predict the virus-host interactions. Both HostG [22] and CHERRY [23] construct knowledge graph and apply graph convolutional neural networks (GCNs) [24] for prediction. HostG also regards the prediction task as a multi-classification problem while CHERRY utilizes graph autoencoder architecture and considers the prediction task as link prediction task which take the host with the highest predicted score as the result.

In this study, we propose a novel method called PHPGCA where we approach the host prediction task as a recommendation task, aiming to recommend the host with the highest probability for specific viruses. Recognizing that traditional supervised-learning formulations may suffer from the lack of labeled interactions between viruses and hosts, we leverage the implicit information from unlabeled interactions to solve the host prediction task. Moreover, we introduce an auxiliary self-supervised task to further enhance the robustness of our model. This auxiliary task generates multiple representation views with noise, maximizing the consistency between different perspectives of a particular node compared to those of other nodes with graph contrastive augmentation. To optimize both the semi-supervised and self-supervised tasks, we employ a multi-task training strategy. Our approach is compared with state-of-the-art methods, and the results demonstrate the superior performance of our model in host prediction accuracy.

## Materials and methods

### Datasets

The evaluation of the performance of different methods is conducted on CHERRY dataset [23]. We download the viruses and prokaryotes from the github repository, containing 1875 viruses. The CHERRY dataset can be split into training and testing data according to the provided raw files, encompassing 1260 positive pairs for training and 615 positive pairs for testing. The training dataset comprises viruses from 174 distinct species, whereas the testing dataset encompasses viruses from 88 distinct species. Notably, there is an overlap of 56 species between the training and testing datasets.

### PHPGCA model

**Fig 1:**
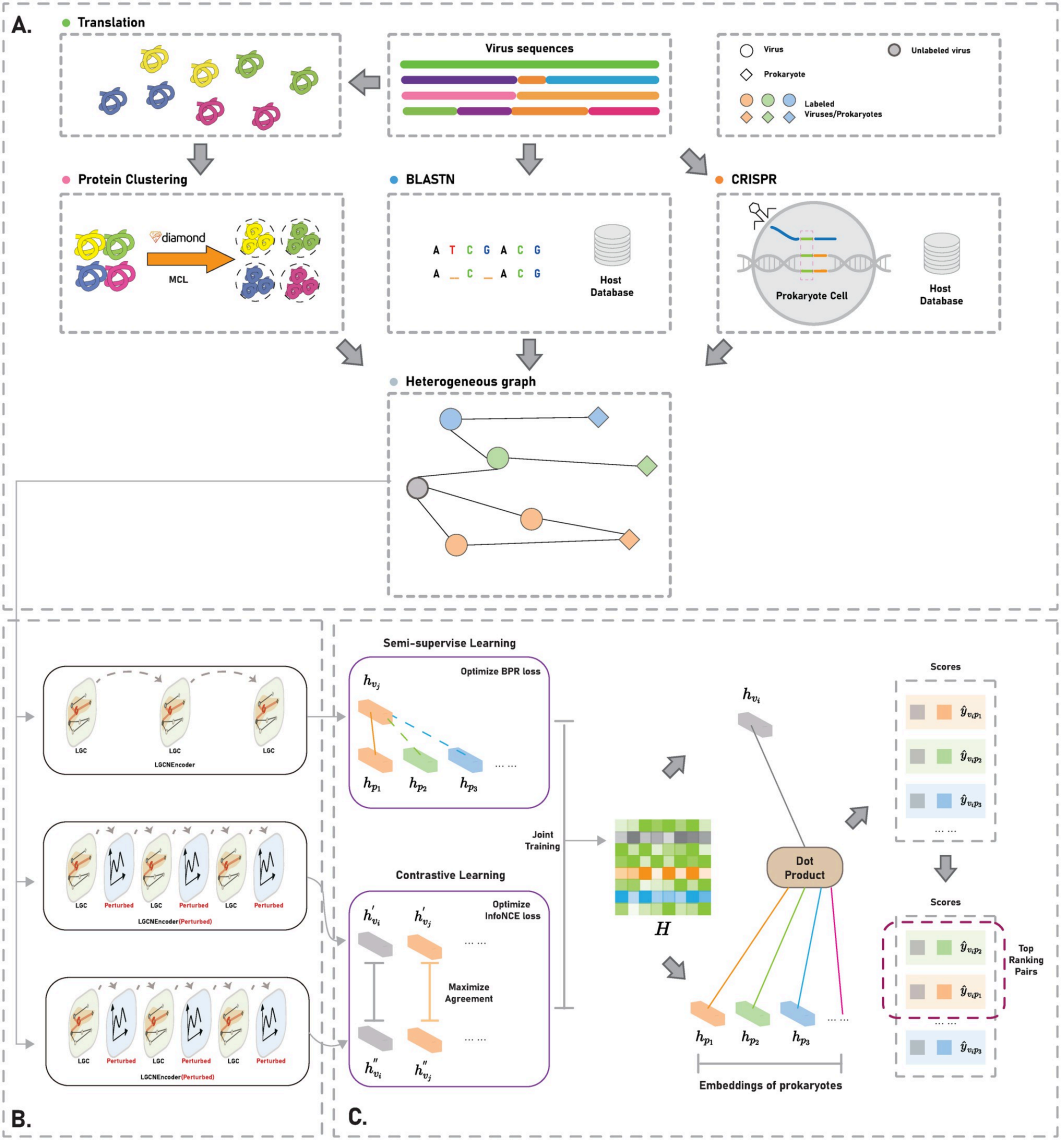
Overall framework illustration of our proposed PHPGCA. (A) Construction of the heterogeneous graph, containing virus-virus and virus-prokaryote edge construction. (B) The architecture of Light Graph neural network with contrastive augmentation. (C) Model training and host range prediction by ranking the scores in descending order.

In the following sections, we will explain our framework in meticulous detail. Our framework is bifurcated into three distinct components. The first part entails the construction of a phage-prokaryote heterogeneous graph, while the second part involves the utilization of graph encoder to encode the embeddings of nodes. The final component of our framework revolves around the application of a multi-task training strategy. This strategy involves training the model using multiple tasks, combining semi-supervised learning and self-supervised learning techniques. Through this training process, we enhance the model’s ability to predict the host of phages accurately. Fig 1 provides a visual depiction of the framework, serving as an illustrative guide to its underlying structure and processes.

#### Construction of the heterogeneous graph

As the initial step in our framework, we focus on the construction of a heterogeneous graph that incorporates both viruses and prokaryotes. This graph serves as a representation of the potential topology and relationships between viruses and prokaryotes, allowing us to capture comprehensive features. To be more precise, the heterogeneous graph *G* = (*O*, *E*) is composed of nodes *o*_*i*_ ∈ *O*, where *i* = 1, 2, …, *M*. Each edge between *o*_*i*_ and *o*_*j*_ is denoted as a tuple (*o*_*i*_, *o*_*j*_)∈*E*. The graph consists of two types of nodes: viral nodes $v_{i}\in V$ and prokaryotic nodes $p_{i}\in P$.

The connections between viruses and prokaryotes are the main links in the virus-prokaryote heterogeneous graph. We adhere to the graph construction method outlined in CHERRY [23] to construct the heterogeneous graph. Fig 1A provides an illustration of the components involved in building the heterogeneous graph.

(1)Edge construction of virus-virus

Protein plays a pivotal role in the biological composition of organisms and serves as a crucial benchmark for assessing functional similarity among different species. Following the approach in [23] and [22], we establish connections between different viruses. Given two viruses, denoted as *A* and *B*, the probability of these viruses sharing at least c common protein clusters is calculated using Eq (1).

$$\begin{array}{c} P\left(\geq c\right)=\sum_{i=c}^{min\left(a,b\right)}\frac{\left(\begin{array}{c} a\\ i \end{array}\right)\left(\begin{array}{c} n-a\\ b-i \end{array}\right)}{\left(\begin{array}{c} n\\ b \end{array}\right)} \end{array}$$ (1)

*a* and *b* are the numbers of proteins contained in *A* and *B*. Based on the assumption that *A* and *B* share *c* common protein clusters with different hosts, the probability *P* is hoped to be smaller than a cutoff and we followed [23] to link two viruses if the probability is smaller than *τ*_1_:

$$\begin{array}{c} virus-virus=\{\begin{array}{c} 1\;if\;P<\tau_{1}\\ 0\;otherwise \end{array} \end{array}$$ (2)

(2)Edge construction of virus-prokaryote

We then construct the virus-prokaryote edges with three different types of links: CRISPR, BLAST and the established interactions derived from training data. The parameter selection aligns with the methodology described in CHERRY [23].

Eq (3) represents the formulation of edge construction for virus-prokaryote connections in the heterogeneous graph. Based on the three types of links (CRISPR-based, BLASTN-based, and known dataset-based), the edges between specific viruses and prokaryotes are created in the graph.

$$\begin{array}{c} virus-prokaryote=\{\begin{array}{c} 1\;if\;CRISPR\;alignment\\ 1\;if\;BLASTN\;E_{value}<\tau_{2}\\ 1\;if\;Known\;interaction\\ 0\;otherwise \end{array} \end{array}$$ (3)

#### Light graph neural network with contrastive augmentation

We utilize the virus-prokaryote heterogeneous graph constructed above to predict the host using the graph contrastive learning model. The main task pipeline involves parameterizing the two distinct types of nodes, viruses and prokaryotes, as embeddings. These embedding parameters are learned using traditional machine learning techniques or deep learning methods. The learned embeddings are then utilized to compute the scores through dot product. In our proposed approach, we utilize a graph convolutional encoder to obtain embeddings for the virus and prokaryote nodes in the heterogeneous graph. During the model parameter learning process, contrastive learning aids in augmenting the available data without requiring additional labels. Subsequently, embeddings for all nodes in the graph are learned, enabling calculation of prediction scores between a specific virus and all prokaryotes.

(1)LGCN Encoder

Graph neural networks(GNNs) are powerful architectures for semi-supervised learning on graph-structured data. The basic idea of GNNs is to learn the representations for nodes by aggregating the information of nodes. Almost all variants of GNNs include neighborhood aggregation operation:

$$\begin{array}{c} h_{v}^{\left(k+1\right)}=AGGR\left(h_{v}^{\left(k\right)},h_{p}^{\left(k\right)}:p\in N_{v}\right) \end{array}$$ (4)

where $h_{v}^{\left(k\right)}$ and $h_{p}^{\left(k\right)}$ denote the representation of the nodes after *k* layers propagation in the graph. $N_{v}$ denotes the set of nodes that are interacted with node *v*. The *AGGR* function controls how to utilize the representations. For example, the most prevalent GNN model Graph Convolutional Network(GCN) summarizes the features of neighbors and transforms them by weight matrices and nonlinear activation to obtain the new representation of a target node. However, not all applications require complex GNN model and the simplified model LightGCN(LGCN) [25] is chosen to be the backbone of our model.

LGCN is initially proposed to address the challenges in recommender systems by simplifying the graph model architecture to alleviate training difficulties. In contrast to conventional recommendation tasks that commonly utilize bipartite graphs, our graph encompasses a more intricate structure by encompassing virus-virus connections in addition to virus-prokaryote connections. This deliberate design choice enables us to comprehensively capture a broader spectrum of relationships and interactions within the phage-host ecosystem, thereby advancing our understanding of its complexity. Light Graph Convolution(LGC) and Layer Combination are the key components of LGCN. LGC simplified the *AGGR* function of the neighborhood by discarding sophisticated transformation and is defined as:

$$\begin{array}{c} h_{v}^{\left(k+1\right)}=\sum_{w\in N_{v}^{v2v}}Norm_{vw}^{v2v}h_{w}^{\left(k\right)}+\sum_{p\in N_{v}^{v2p}}Norm_{vp}^{v2p}h_{p}^{\left(k\right)} \end{array}$$ (5)

$$\begin{array}{c} h_{p}^{\left(k+1\right)}=\sum_{p\in N_{p}^{p2v}}Norm_{pv}^{p2v}h_{v}^{\left(k\right)} \end{array}$$ (6)

$$\begin{array}{c} Norm_{vw}^{v2v}=\frac{1}{\sqrt{\left|N_{v}^{v2v}\right|+\left|N_{v}^{v2p}\right|}\sqrt{\left|N_{w}^{v2v}\right|+\left|N_{w}^{v2p}\right|}} \end{array}$$ (7)

$$\begin{array}{c} Norm_{vp}^{v2p}=\frac{1}{\sqrt{\left|N_{v}^{v2p}\right|+\left|N_{v}^{v2v}\right|}\sqrt{\left|N_{p}^{p2v}\right|}} \end{array}$$ (8)

$$\begin{array}{c} Norm_{pv}^{p2v}=\frac{1}{\sqrt{\left|N_{p}^{p2v}\right|}\sqrt{\left|N_{v}^{v2p}\right|+\left|N_{v}^{v2v}\right|}} \end{array}$$ (9)

Where *v* and *w* are viral nodes, while *p* represents a prokaryotic node belonging to P. $N_{v}^{v2v}$ indicates the neighbouring viral nodes connected with *v*. Similarly, $N_{v}^{v2p}$ refers to the neighbouring prokaryotic nodes connected with *v*, and $N_{p}^{p2v}$ represents the neighbouring viral nodes connected with *p*. The normalization term *Norm* helps to prevent the graph from scaling via graph convolution operations. Due to the difference in the layer combination, LGC abandons the operation of aggregating the target node itself. Instead of only using the embeddings from the last layer, combining embeddings from each layers contains more information. The trainable parameters are the embeddings of viruses and prokaryotes, which are used to be the embeddings at the 0-th layer. Once the initial embeddings are given, the embeddings of different layers can be computed by the LGC. We obtain the final embeddings by aggregating each layer and defined as:

$$\begin{array}{c} h_{v}=\frac{1}{K}\sum_{k=1}^{K}h_{v}^{\left(k\right)} \end{array}$$ (10)

$$\begin{array}{c} h_{p}=\frac{1}{K}\sum_{k=1}^{K}h_{p}^{\left(k\right)} \end{array}$$ (11)

Where *K* denotes the number of graph convolution layers.

The LGC can reduce the oversmoothing problem by simplifying the operation of aggregating while the layer combination strategy can capture semantics from different layers, making the representation more powerful. Considering the whole graph, the model of the matrix form can extend from the message passing form. Let the virus-virus interaction matrix be $R_{vv}\in R^{V\times V}$ and virus-prokaryote interaction matrix be $R_{vp}\in R^{V\times P}$, where *V* and *P* denote the number of viruses and prokaryotes in the heterogeneous graph. The value of *R*_*vw*_ is 1 if there is a connection between virus *v* and *w* otherwise 0, while *R*_*vp*_ is 1 if virus *v* has interaction with prokaryote *p*. The adjacency matrix of the heterogeneous graph can be denoted as:

$$\begin{array}{c} A=(\begin{array}{cc} R_{vv} & R_{vp}\\ R_{vp}^{T} & 0 \end{array}) \end{array}$$ (12)

$$\begin{array}{c} D_{ii}=\sum_{j}A_{ij} \end{array}$$ (13)

**D** is the degree matrix and **D**_*ii*_ denotes the number of interactions of the node *i* calculated via Eq (13). The matrix form of LGC can form as:

$$\begin{array}{c} H^{\left(k+1\right)}=\left(D^{-\frac{1}{2}}AD^{-\frac{1}{2}}\right)H^{\left(k\right)} \end{array}$$ (14)

Where the embedding matrix of 0-th layer $H^{\left(0\right)}\in R^{(V+P)\times C}$. The final embeddding matrix can be computed as:

$$\begin{array}{c} H=\frac{1}{K}\left(\tilde{A}H^{\left(0\right)}+...+{\tilde{A}}^{K}H^{\left(0\right)}\right) \end{array}$$ (15)

Where $\tilde{A}=D^{-\frac{1}{2}}AD^{-\frac{1}{2}}$. We use Xavier uniform initialization [26] to randomly init the embeddings of virusesand prokaryotes nodes and set the dimension size to 128.

The LGCN encoder with perturbation introduces slight differences compared to the original LGCN encoder. The perturbed version adds noise to each hidden layer, resulting in the generation of different representation views for graph contrastive augmentation. The upcoming section will provide a detailed description.

(2)Graph contrastive augmentation

Due to the issue of data sparsity in biological data, supervised learning methods may ignore much of the information present in the raw data. Contrastive learning (CL) [27] can address this limitation by extracting general representations from massive unlabeled data without requiring annotations, thereby serving as an auxiliary technique to enhance existing models and make them more robust. The core idea of CL is to augment data by leveraging representational invariances. In the case of GNN architectures, dropout of nodes or edges is commonly used to create diverse graphs and improve the generalization capability of graph models. However, applying CL by perturbing the graph structure can be time-consuming and challenging to manipulate. In our approach, inspired by [28], we focus on the embedding space for contrastive learning. Given a node v, different augmentation views of the node form as:

$$\begin{array}{c} h_{v}^{{}^{'}}=h_{v}+\psi_{v}^{{}^{'}},\;h_{v}^{{}^{''}}=h_{v}+\psi_{v}^{{}^{''}} \end{array}$$ (16)

where $\psi_{v}^{{}^{'}}$ and $\psi_{v}^{{}^{''}}$ are the different noise vectors. The noise vectors assigned to individual nodes and layers are distinct, aiming to generate diverse noise and subsequently employ contrastive learning to enhance the robustness of node embeddings. The modulus of the noise vectors ‖*ψ*‖_2_ = *ϵ* controls the intensity. $\psi =\bar{\psi }⊙sign\left(h_{i}\right),\;\bar{\psi }\in R^{d}∼U\left(0,1\right)$ controls the range of augmented embeddings, which will not create much disturbance, remaining much information from the origin embeddings. The final embeddings are computed as:

$$\begin{array}{c} \begin{array}{cc} H^{{}^{'}}= & \frac{1}{K}(\left(\tilde{A}H^{\left(0\right)}+\psi^{\left(1\right)}\right)+...\\ & +({\tilde{A}}^{K}H^{\left(0\right)}+{\tilde{A}}^{K}\psi^{\left(1\right)}+...+\tilde{A}\psi^{\left(K-1\right)}+\psi^{\left(K\right)})) \end{array} \end{array}$$ (17)

We follow [28] and skip the input embeddings **H**^(0)^ to achieve better performance, The operation of perturbing the origin embeddings from each layer is easier to manipulate than dropping out nodes or edges from the graph.

#### Model training

We leverage a multi-task training strategy to jointly optimize the semi-supervised learning task and self-supervised learning task, as defined in Eq (18)

$$\begin{array}{c} L=L_{main}+\lambda L_{aux} \end{array}$$ (18)

Where $L_{main}$ refers to the training loss of the main task, while λ is a parameter that determines the magnitude of the contrastive learning task. Additionally, $L_{aux}$ refers to the training loss of the auxiliary task.

The host prediction for viruses serves as the main task, while the contrastive learning task is utilized as an auxiliary approach to augment data. The trainable parameters of the graph model are limited to the embeddings of viruses and prokaryotes in the 0-th layer. To optimize these parameters, previous approaches typically treat it as a supervised learning task, where the supervision signal is derived from the observed interactions or the linked edges in the graph.

We utilize the Bayesian Personalized Ranking (BPR) loss with negative sampling to optimize the semi-supervised learning task. The BPR loss is commonly used in CF models, as it encourages the prediction scores of observed interactions to be higher than those of unobserved pairs:

$$\begin{array}{c} L_{main}=-\sum_{v=1}^{V}\sum_{p\in N_{v}^{v2p}}ln\sigma \left({\hat{y}}_{vp}-{\hat{y}}_{vq}\right) \end{array}$$ (19)

Where $\;{\hat{y}}_{vp}=h_{v}^{T}h_{p}$ is the preference score. *σ*(⋅) is the sigmoid function. *q* is the prokaryote node sampled from the unobserved connection of virus node *v*. For the self-supervised auxiliary task, we do not require additional label annotations. Instead, we utilize the two views of nodes in the heterogeneous graph. For the same node, we treat the views as positive pairs, while negative pairs are formed with different nodes. The positive pairs encourage consistency between different views of the same node with perturbation, while also strengthening the distinction between different nodes. Following the approach proposed in [28], we adopt the InfoNCE Loss [29] to optimize the self-supervised augmentation task. This involves minimizing the agreement of negative pairs and maximizing the agreement of positive pairs, without the need for additional label annotations:

$$\begin{array}{c} L_{aux}=\sum_{i\in N}-log\frac{exp(h_{i}^{{}^{'}T}h_{i}^{{}^{''}}/\rho )}{\sum_{j\in N}exp\left(h_{i}^{{}^{'}T}h_{j}^{{}^{''}}/\rho \right)} \end{array}$$ (20)

where *N* represents the node set of the heterogeneous graph, and *ρ* > 0 (e.g., 0.2) is the temperature hyperparameter. We optimize the model using the Adam optimizer [30] with a learning rate of 0.001. Following the approach proposed in [28], we choose the temperature *ρ* = 0.2, which has been reported as the optimal hyperparameter for the contrastive augmentation task.

Once the model is trained, each node in the heterogeneous graph is assigned a final embedding. These embeddings capture the representations of the nodes in a lower-dimensional space, encoding their characteristics and relationships. To predict virus-host interactions, we calculate the scores between the test virus nodes and all prokaryote nodes using a dot product operation, as described in the model training section. These scores reflect the similarity or affinity between the viruses and prokaryotes. By sorting the scores in descending order, we can identify the top scoring pairs, which correspond to the most likely virus-host interactions.

## Results

### Evaluation criteria

To compare our model with state-of-the-art methods, we use the accuracy metric to evaluate the performance which is shown as Eq (21):

$$\begin{array}{c} Accuracy=\frac{number\;of\;correct\;predictions}{number\;of\;prediction\;samples} \end{array}$$ (21)

The host prediction for the specify virus is correct if the taxon of predicted prokaryote is same as the known interaction.

### Performance comparison of different neural network-based methodologies

**Fig 2:**
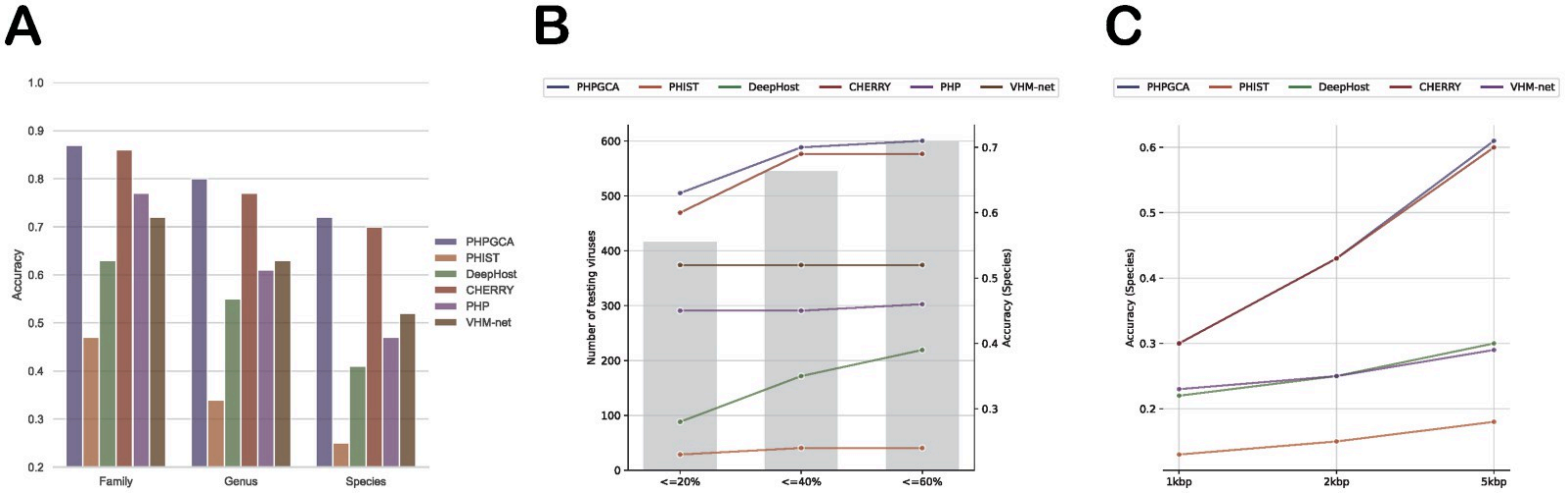
Performance on CHERRY dataset. (A) The results on CHERRY dataset, from the species level to the family level, are presented from left to right. (B) Performance of different similarity between training set and testing set at tht species level. X-axis: dashing similarity threshold. Left Y-axis: number of test viruses under given threshold. Right-axis: Accuracy at species level. (C) Performance evaluation of contigs of varying lengths at the species level. X-axis: length of contigs. Y-axis: Accuracy on species level.

In the host prediction experiment, we use the CHERRY dataset mentioned above to evaluate the performance of our model and compare it with other state-of-the-art host prediction tools, containing PHIST [17], PHP [18], DeepHost [20], VHM-net [21], CHERRY [23], ranging from species to family level. For DeepHost and PHP, we retrain the models using the given hyperparameters in their respective papers to adapt to the datasets. For CHERRY, we reconstruct the knowledge graph and retrain the model. As for other learning methods, since they are difficult to retrain, we use their pre-trained models to evaluate their performance. As shown in Fig 2, our approach outperforms other state-of-the-art methods on CHERRY dataset, from family level to species level. In the case of the CHERRY dataset, PHPGCA outperforms the second-best method, CHERRY, by a margin of 2%. It is observed that the prediction accuracy improves as we move from family to species level, as features with more information from higher taxonomic rankings are easier to distinguish.

#### Performance on different similarity between training and testing set

We employ the dashing [31] algorithm to compute the similarity between the training set and the testing set. For each test virus, we calculate the similarity between all the training viruses and select the maximum similarity value as an indicator of how closely the test virus resembles the training set. Subsequently, we segment the test viruses into different similarity threshold categories and evaluate accuracy on the species level. Fig 2B depicts the outcomes, revealing that as the similarity between the training and testing sets becomes more pronounced, the performance of all methodologies exhibits an upward trend. Importantly, PHPGCA continues to demonstrate superior performance in comparison to other methods.

#### Performance on different length contigs

Initially, we undertake the creation of contigs of varying lengths by randomly segmenting viral contigs into three specific lengths: 1kbp, 2kbp, and 5kbp. For every test virus across each length category, we carry out a randomized division of the contigs of the specified lengths. This process is reiterated ten times to ensure robustness. In the case of 1kbp and 2kbp conditions, a cumulative sum of 6150 contigs is generated, whereas the 5kbp condition yields 6140 contigs due to certain virus sequences in the testing set being insufficient to meet the 5kbp criterion. We proceed to juxtapose our method with PHIST, DeepHost, CHERRY, and VHM-net, all of which possess the capability to predict hosts at the species level. The outcomes, illustrated in Fig 2C, indicate a discernible trend wherein the performance of all methods improves as the contig length increases. Remarkably, PHPGCA continues to outperform other methods across various contig lengths.

### Performance comparison on human gut phage dataset

**Fig 3:**
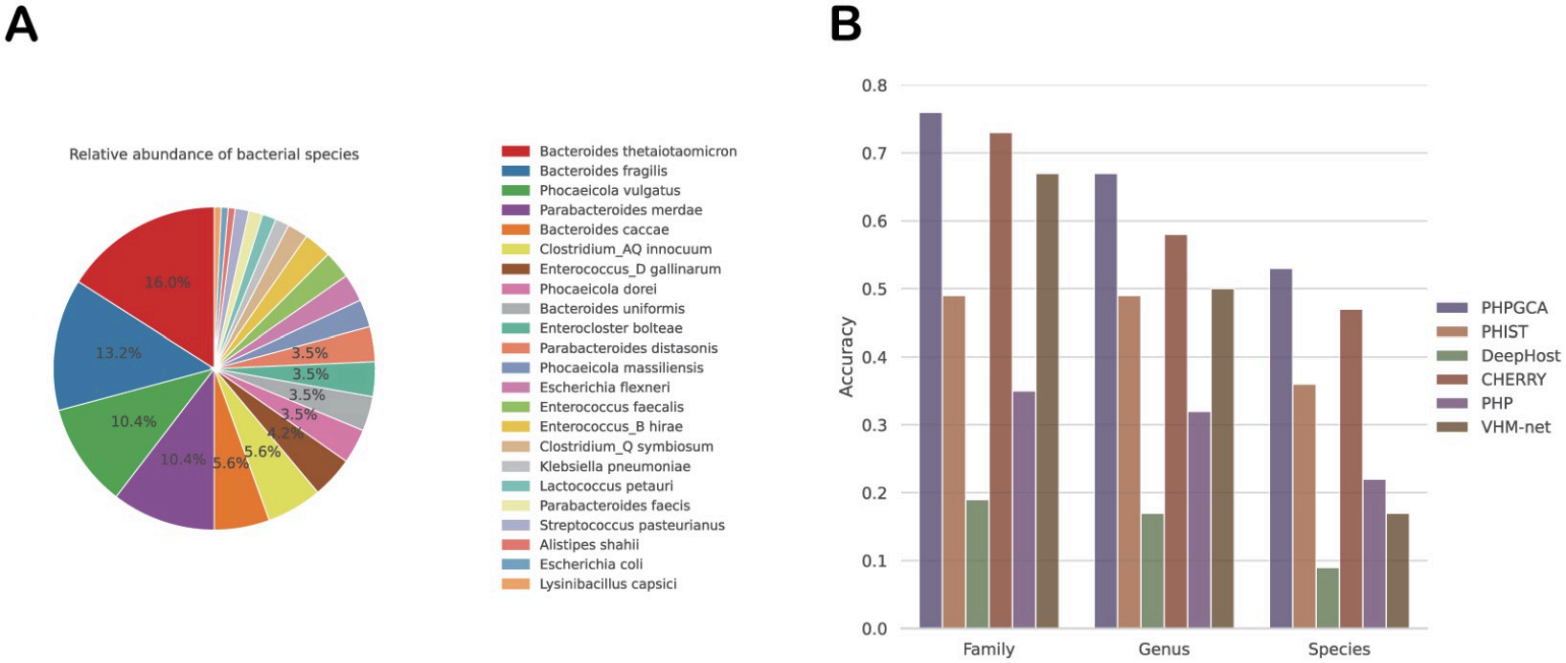
The experimental result on the GPIC dataset. (A): The distribution of different species. (B): Performance of host prediction compared with the state-of-the-art methods from family to species level.

Bacteriophages are plentiful in the human gut. As predators of bacteria, phages have a significant impact on both the composition and function of the human gut microbiome. Furthermore, the use of phage isolates targeted towards gut bacteria has shown promise as a powerful tool for manipulating the microbiome. In several cases [32–34], the application of specific phages has proven effective in eliminating pathogens, leading to favorable outcomes. These successful interventions highlight the feasibility of utilizing phages for engineering the gut microbiome. Nevertheless, the lack of universally applicable marker genes and the significant sequence variation observed among phage genomes pose challenges. Consequently, a large proportion of potential bacterial phage sequences identified from metagenomic data obtained from the human gut (ranging from 75% to 99%) cannot be taxonomically classified or associated with specific microbial hosts [35]. In a recent publication by Shen et al. [36], they introduced a comprehensive collection of gut phage isolates known as the Gut Phage Isolate Collection (GPIC). This collection was established through the utilization of the soft agar overlay method, which enabled the successful isolation and purification of phages. Remarkably, the authors were able to isolate phages that specifically targeted 42 different bacterial species found in the human gut. These bacterial species encompassed a diverse range, including 15 species from the Bacteroidetes phylum, 19 species from Firmicutes, 4 species from Actinobacteria, and 4 species from Proteobacteria. This achievement highlights the significant progress made in understanding the phage-bacteria interactions within the human gut ecosystem. Combining with the prokaryotes from the supplementary data and CHERRY database, we extracted 144 phages to validate the performance of our model. Fig 3A shows the ditribution of phage target host on species level. We compared the performance of PHPGCA with state-of-the-art methods using the 144 phages extracted from GPIC, and found that PHPGCA still outperformed other methods. In fact, it achieved 6%, 9%, and 3% higher accuracy than the second-best method CHERRY at the levels of species, genes, and family, respectively.

### Host prediction on metagenomic data

In this section, we validate the performance of our model in host prediction for putatively novel viruses identified from metagenomic data. Since metagenomic data can contain numerous distinct species or components, it is essential to use prokaryotic virus identification tools to screen viral contigs from metagenomic data before applying our model for host prediction. As an example, widely used tools such as Metaviral spades [37], Seeker [38], and VirSorter [39] can be employed for virus identification.

**Fig 4:**
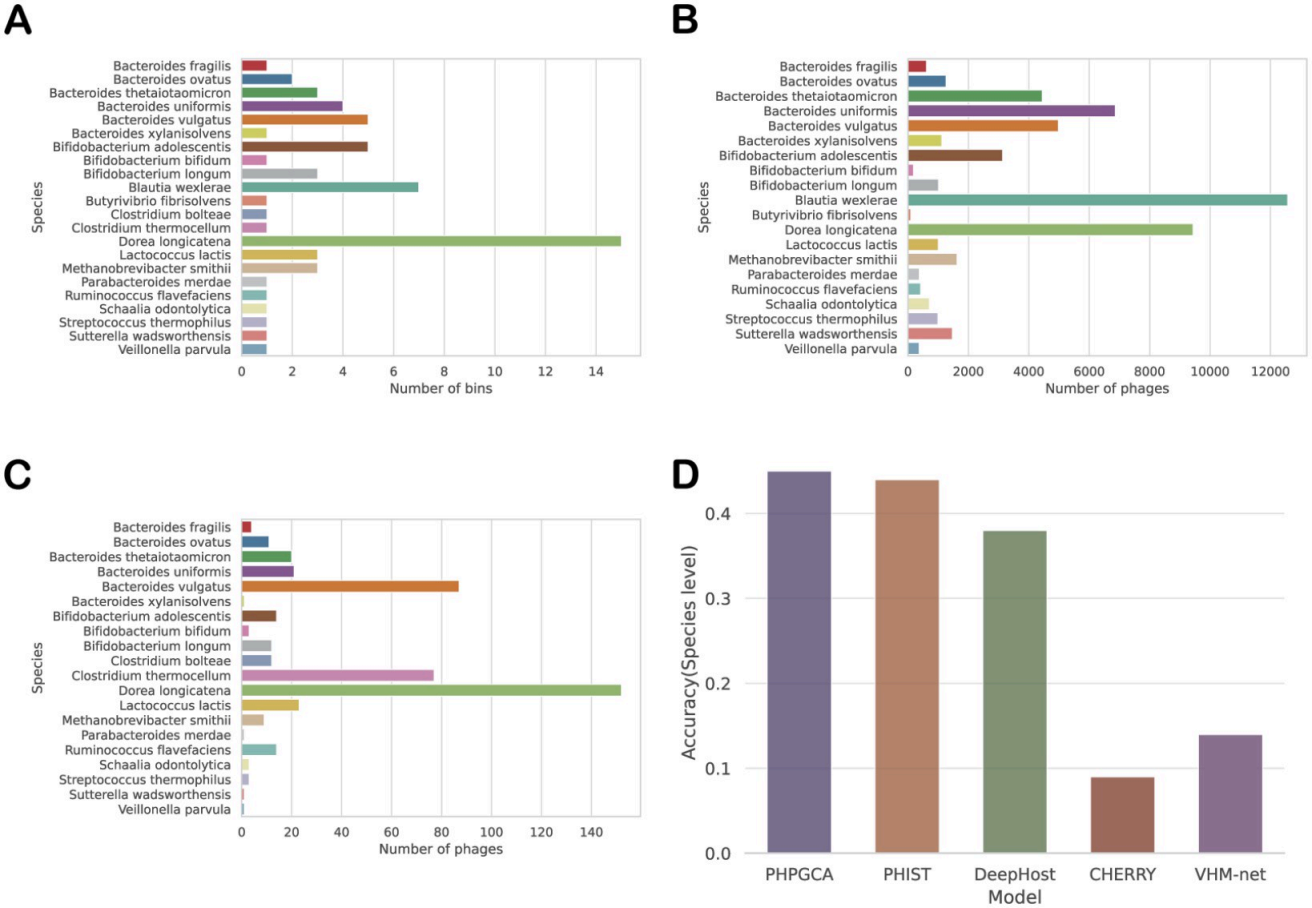
The description of Hi-C sequencing dataset and host prediction performance. (A): The number of bins corresponding to different species. (B): The number of training contigs corresponding to different species. (C): The number of testing contigs corresponding to different species. (D): The performance of host prediction on testing data.

In our experiment, we utilize MetaHiC [40], a method that detects interactions between phages and assembled bacterial genomes in human gut samples. MetaHiC captures DNA-DNA collisions that occur during phage replication inside bacterial cells, providing a high-quality benchmark for host prediction. We use the phage-bacteria interactions provided by MetaHiC as the ground truth to evaluate the performance of current state-of-the-art methods at the species level. To obtain the necessary data for our evaluation, we use the supplementary data from MetaHiC, which provides bins or contigs and their corresponding taxonomy information. However, it should be noted that only 62 bins have species taxon annotations available. Therefore, we extract contigs from these bins to train and test our model. As shown in Fig 4, our approach still behaves competitive on metagenomic data compared to the state-of-the-art methods. PHPGCA achieved an accuracy rate of 46%, surpassing PHIST by 2%.

### Multi-host prediction

Determining whether phages have the ability to infect multiple hosts is important for various reasons, including guiding phage therapy, aiding bacterial identification, understanding phage ecology and evolution, and facilitating biotechnological applications. It provides valuable information for the field of phage research and has practical applications in various fields, including medicine, microbiology, ecology, and food safety.

#### Case study one: Multi-host prediction of bacteriophage phi92

Bacteriophage phi92 is a large, lytic myovirus that was initially isolated from pathogenic Escherichia coli strains carrying a polysialic acid capsule in 1983. However, further investigation showed that its host range is not limited to polysialic acid-encapsulated E. coli strains, but also includes various laboratory strains of E. coli and many Salmonella strains. In a study by Schwarzer et al. [41], the host specificity of phi92 was re-examined by testing it on multiple bacterial strains, including laboratory strains of E. coli and a wide range of Salmonella strains. The results demonstrated that bacteriophage phi92 can infect both E. coli and Salmonella strains, as evidenced by their respective plating efficiencies.

**Fig 5:**
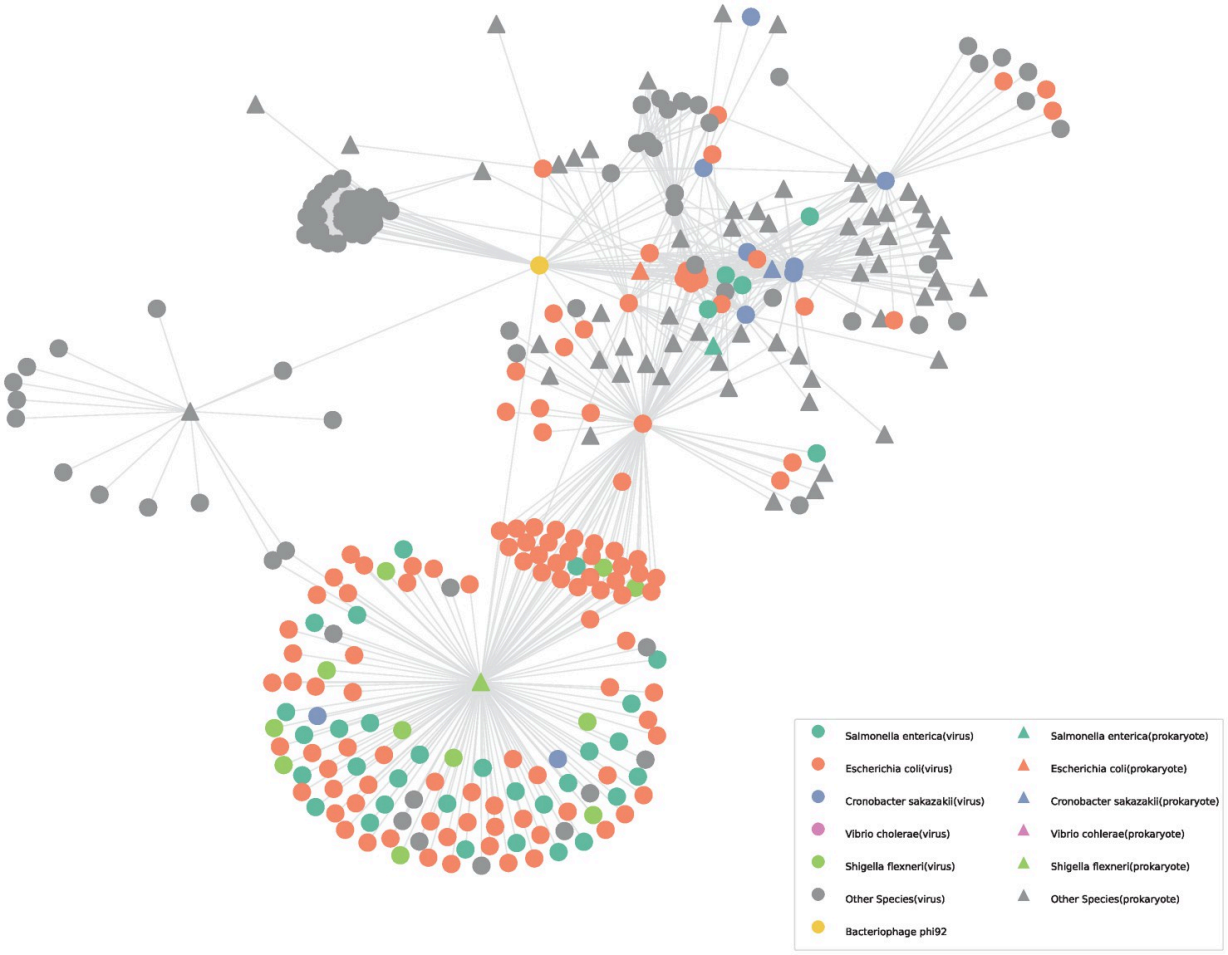
Visualization of graph of second-order neighbors of bacteriophage phi92. The nodes are colored based on their respective labels. For prokaryotic nodes, the labels represent their species, whereas for virus nodes, the labels represent their hosts’ species. In the visualization, the top 5 species that were predicted are shown in distinct colors, while all other nodes are represented in gray.

Fig 5 illustrates the second-order neighbors of bacteriophage phi92. In this network, the neighbors include various labels, encompassing not only the true host that phi92 precisely infected but also other labels represented by the grey-colored nodes. Predicting the accurate label for phi92 using conventional label propagation methods based on the heterogeneous graph can be challenging. However, PHPGCA demonstrates the capability to accurately predict the host of phi92 even in this scenario. We use PHPGCA to identify potential hosts infected by bacteriophage phi92. The prediction scores were sorted in descending order, and the top 5 scores were considered as the predicted host range. Table 1 displays the top 5 predictions obtained from our analysis. The outcomes of our analysis reveal that PHPGCA successfully predicted Escherichia coli and Salmonella enterica as the potential hosts for bacteriophage phi92.

**Table 1:** Top 5 host prediction of bacteriophage phi92.

Rank	Species	Score
1	***Salmonella enterica***	0.997
2	***Escherichia coli***	0.987
3	Cronobacter sakazakii	0.979
4	Vibrio cholerae	0.978
5	Shigella flexneri	0.971

Bold in italics indicates a species with accurate predictions.

#### Case study two: Multi-host prediction of phages on food safety

Globally, there are approximately 600 million reported cases of foodborne illnesses each year, resulting in the unfortunate loss of 420,000 lives [42]. Among the various pathogens responsible for foodborne illnesses, Escherichia coli, Salmonella enterica and Shigella flexneri are recognized as significant contributors to these outbreaks. These two bacterial species are widely acknowledged as major causes of foodborne illnesses, posing a significant threat to public health and food safety worldwide. Phages, characterized by their high diversity, have the unique capability to infect and lyse host bacteria, leading to the release of progeny phages that can initiate subsequent infections. This ability of phages to target and destroy bacterial pathogens has been harnessed and exploited for food safety purposes. The antimicrobial activities of phages have proven valuable in mitigating the risk of foodborne illnesses caused by bacteria such as Escherichia coli and Salmonella enterica. By specifically targeting and eliminating these bacterial pathogens, phages offer a promising approach to enhance food safety and reduce the incidence of foodborne illnesses.

We collect three phages, namely HY01 [43], EscoHU1 [44] and LPEK22 [45], that have demonstrated potential in inhibiting the growth of Escherichia coli, Salmonella enterica and Shigella flexneri in food. Table 2 presents the host range and the strain used for isolating the three phages. We have utilized the PHPGCA to predict the potential host range of HY01, EscoHU1, and LPEK22. Table 3 presents the top 5 prediction scores obtained from PHPGCA, arranged in descending order. The results demonstrate that PHPGCA successfully predicted the multi-species targeted by the three phages, as all of them are included in the predictions. This highlights the accurate predictive capabilities of PHPGCA for determining the potential hosts of phages infecting multiple species.

**Table 2:** Host range on species level and the host for phage isolation of three phages.

Phage	Species	Isolate strain
HY01	Escherichia coli	*E.coli* O157:H7 ATCC 43890
	Salmonella enterica	
EscoHU1	Escherichia coli	*E.coli* O157:H7 RIMD 0509939
	Shigella flexneri	
LPEK22	Escherichia coli	*E.coli* LEC8
	Salmonella enterica	
	Shigella sonnei	

**Table 3:** Top 5 host prediction of three phages.

Phage	Species				
	Rank1	Rank2	Rank3	Rank4	Rank5
HY01	***Escherichia coli***	***Salmonella enterica***	Shigella flexneri	Klebsiella pneumoniae	Shigella boydii
EscoHU1	***Escherichia coli***	Salmonella enterica	***Shigella flexneri***	Shigella boydii	Shigella sonnei
LPEK22	***Escherichia coli***	***Salmonella enterica***	Shigella flexneri	Shigella boydii	***Shigella sonnei***

Bold in italics indicates a species with accurate predictions.

### Ablation study

**Fig 6:**
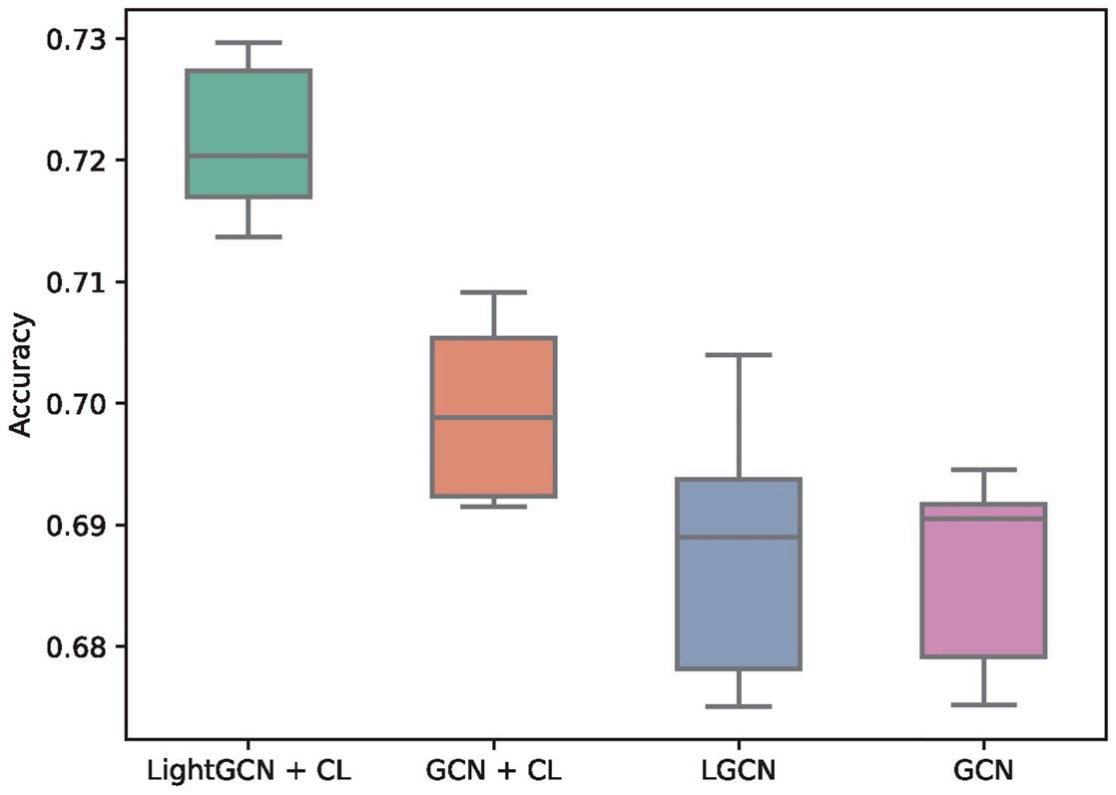
Host prediction accuracy with diffrenet ablation combinations.

In our ablation studies, we investigate the impact of different graph convolutional operations and the effectiveness of contrastive augmentation. As shown in Fig 6, our evaluation encompasses several components, including the use of different GCN models and the incorporation of contrastive learning. We perform comparisons involving LightGCN, the graph neural network architecture employed in our proposed model, and GCN, a commonly used graph neural network. Additionally, we assess the performance of LightGCN and GCN when combined with contrastive augmentation.

The experimental results show that the best performance is achieved by the LightGCN graph architecture with contrastive augmentation. This is followed by the combination of GCN with contrastive augmentation. The results suggest that contrastive augmentation enhances the prediction ability of both LightGCN and GCN, without requiring additional annotation data. Without augmentation, the performance of LightGCN and GCN is inferior. Overall, the key components of our model: LightGCN and contrastive augmentation, both contribute to improving the prediction ability in the host prediction task.

### The impact of hyperparameters

**Fig 7:**
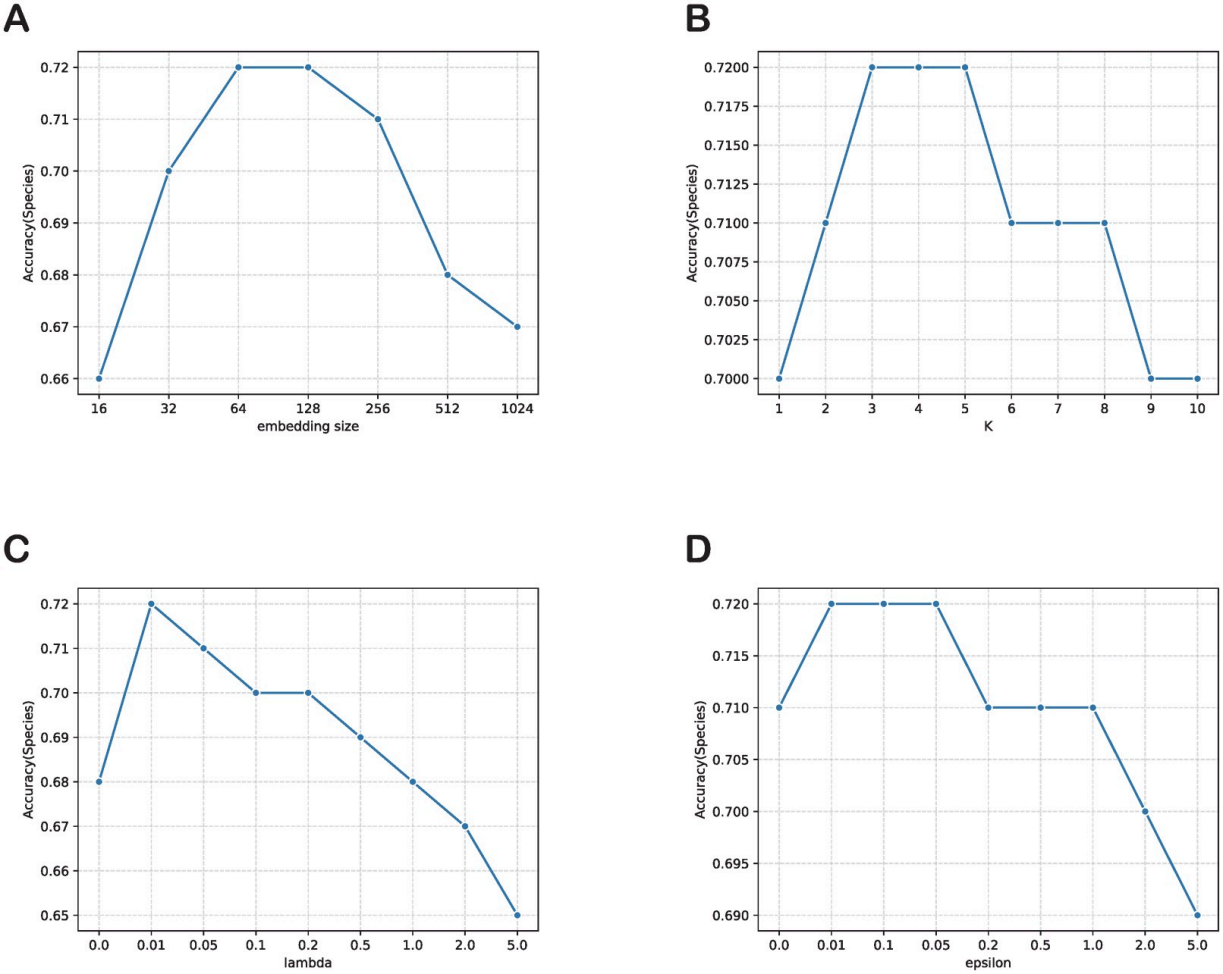
The effect of hyperparameters on the host prediction performance. (A): Impact of the embedding size. (B): Impact of number of layers. (C): Impact of strength of CL. (D): Impact of magnitude of noise.

In this section of our research, we conduct experiments with different hyperparameters to examine their impact on the performance of our proposed model. The hyperparameters include the embedding size *d*, number of layers *K*, and the augmentation hyperparameters λ and *ϵ*. Specifically, we fixed other hyperparameters and experimented with different values of embedding size *d* (16, 32, 64, 128, 256, and 512), and then fixed the optimal *d* and tried different number of layers *K* (1, 2, 3, 4, 5, 6, 7, and 8). For the augmentation hyperparameters, we fixed *ϵ* at 0.1 and experimented with different values of λ (0, 0.01, 0.05, 0.1, 0.2, 0.5, 1, 2, and 5) to investigate their impact on model performance. The results of these experiments are shown in Fig 7, providing valuable insights for selecting optimal hyperparameter settings for our model.

1. *Embedding size d.* For the CHERRY dataset, the prediction accuracy all increase from *d* = 16 to *d* = 128. While the embedding size is set small, the model may not be able to represent the full information of the input data. CHERRY dataset achieves best performance when *d* = 128, which can be the appropriate embedding size for the model. However, the prediction ability may decrease if the embedding size is set too large, causing the overfitting problem and reducing model generalization.
2. *Number of layers K.* By fixing *d* = 128, we set the range of *K* from 1 to 10. As shown in Fig 7B, the prediction accuracy is infected by different *K*. For CHERRY dataset, *K* = 3 can perform best. We find that in the first few layers, the performance becomes better because the representation of each node is aggregated more fully, capturing the feature of the global graph structure. However, when the *K* increasingly set large leads to the oversmoothing problem. The nodes of the graph aggregate information from their neighbor too many times and the information from different parts of the graph becomes indistinguishable.
3. *Strength of CL λ*. As shown in Fig 7C, the prediction performance on all the dataset increase in the begining with the increase of *lambda*. The phenomenon is expected because the auxiliary contrastive learning task starts to take effect. The range of peak performance that the model maintains varies across different datasets. However, when *lambda* still increase larger, the accuracy decrease even perform worse than those without augmentation.
4. *Magnitude of noise ϵ*. We change the value of *ϵ* from 0 to 5 shown in Fig 7D. Like λ, the model accuracy increase in the beginning but decrease if the value is set too large. when *ϵ* is near 0.01, the model achieve best performance on CHERRY dataset.

## Discussion

In this study, we propose a novel approach for prokaryotic virus host prediction, where we treat the host prediction task as a recommendation task. We start by constructing a virus-prokaryote heterogeneous graph that integrates abundant information, such as protein similarity between viruses and sequence similarity between viruses and prokaryotes. Subsequently, we employ the graph encoder LightGCN to encode the embeddings of the nodes within the graph. Additionally, we employ a graph contrastive learning method to augment the node representations without requiring additional labels. This is achieved by encouraging representations of the same nodes in two different views to be similar, while representations of different nodes in those views to be distinct. Lastly, we employ a multi-task training strategy to optimize the model for the host prediction task. The main task focuses on predicting the host, while the auxiliary task involves contrastive learning. The training strategy allows us to train the model effectively and achieve accurate host predictions.

The experimental results demonstrate that PHPGCA outperforms state-of-the-art methods on three different datasets. Moreover, when applied to metagenomic data, PHPGCA remains competitive with the current state-of-the-art methods. By utilizing PHPGCA, we can calculate preference scores between viruses and prokaryotes, enabling us to effectively assess the host range of newly discovered phages. We further validate our approach through three case studies, showcasing the effectiveness of PHPGCA in determining the host range of these phages.

Despite the performance improvement of PHPGCA in host prediction, there are areas that can be optimized in future work. Firstly, the construction of an accurate and informative virus-prokaryote heterogeneous graph is crucial for the contrastive learning framework. Currently, only virus-virus and virus-prokaryote interactions are utilized, without incorporating prokaryote-prokaryote interactions. Exploring the incorporation of additional biological features in the graph construction could be a potential avenue for further improvement. Secondly, the generation of different views of nodes for contrastive learning is based on simple strategies. Exploring more powerful data augmentation methods may unlock more potential of unlabeled data in improving the performance of PHPGCA in host prediction.

## References

[1] Williamson KE, Radosevich M, Wommack KE. Abundance and diversity of viruses in six Delaware soils. Applied and environmental microbiology. 2005;71(6):3119–3125. doi: 10.1128/AEM.71.6.3119-3125.2005 15933010 PMC1151856

[2] Koskella B, Meaden S. Understanding bacteriophage specificity in natural microbial communities. Viruses. 2013;5(3):806–823. doi: 10.3390/v5030806 23478639 PMC3705297

[3] Gregory AC, Zayed AA, Conceição-Neto N, Temperton B, Bolduc B, Alberti A, et al. Marine DNA viral macro-and microdiversity from pole to pole. Cell. 2019;177(5):1109–1123. 31031001 10.1016/j.cell.2019.03.040 PMC6525058

[4] Suttle CA. Marine viruses—major players in the global ecosystem. Nature reviews microbiology. 2007;5(10):801–812. doi: 10.1038/nrmicro1750 17853907

[5] Townsend EM, Kelly L, Muscatt G, Box JD, Hargraves N, Lilley D, et al. The human gut phageome: origins and roles in the human gut microbiome. Frontiers in cellular and infection microbiology. 2021; p. 498. doi: 10.3389/fcimb.2021.643214 34150671 PMC8213399

[6] Torres-Barceló C, Hochberg ME. Evolutionary rationale for phages as complements of antibiotics. Trends in microbiology. 2016;24(4):249–256. doi: 10.1016/j.tim.2015.12.011 26786863

[7] Doss J, Culbertson K, Hahn D, Camacho J, Barekzi N. A review of phage therapy against bacterial pathogens of aquatic and terrestrial organisms. Viruses. 2017;9(3):50. doi: 10.3390/v9030050 28335451 PMC5371805

[8] de Jonge PA, Nobrega FL, Brouns SJ, Dutilh BE. Molecular and evolutionary determinants of bacteriophage host range. Trends in microbiology. 2019;27(1):51–63. doi: 10.1016/j.tim.2018.08.006 30181062

[9] Edwards RA, Rohwer F. Viral metagenomics. Nature Reviews Microbiology. 2005;3(6):504–510. doi: 10.1038/nrmicro1163 15886693

[10] Wawrzynczak E. A global marine viral metagenome. Nature Reviews Microbiology. 2007;5(1):6–6. doi: 10.1038/nrmicro1582

[11] Edwards RA, McNair K, Faust K, Raes J, Dutilh BE. Computational approaches to predict bacteriophage–host relationships. FEMS microbiology reviews. 2016;40(2):258–272. doi: 10.1093/femsre/fuv048 26657537 PMC5831537

[12] Johnson M, Zaretskaya I, Raytselis Y, Merezhuk Y, McGinnis S, Madden TL. NCBI BLAST: a better web interface. Nucleic acids research. 2008;36(suppl_2):W5–W9. doi: 10.1093/nar/gkn201 18440982 PMC2447716

[13] Gregory MA, Till R, Smith MC. Integration site for Streptomyces phage *φ*BT1 and development of site-specific integrating vectors. Journal of bacteriology. 2003;185(17):5320–5323. doi: 10.1128/JB.185.17.5320-5323.2003 12923110 PMC180994

[14] Groth AC, Calos MP. Phage integrases: biology and applications. Journal of molecular biology. 2004;335(3):667–678. doi: 10.1016/j.jmb.2003.09.082 14687564

[15] Villarroel J, Kleinheinz KA, Jurtz VI, Zschach H, Lund O, Nielsen M, et al. HostPhinder: a phage host prediction tool. Viruses. 2016;8(5):116. doi: 10.3390/v8050116 27153081 PMC4885074

[16] Ahlgren NA, Ren J, Lu YY, Fuhrman JA, Sun F. Alignment-free oligonucleotide frequency dissimilarity measure improves prediction of hosts from metagenomically-derived viral sequences. Nucleic acids research. 2017;45(1):39–53. doi: 10.1093/nar/gkw1002 27899557 PMC5224470

[17] Zielezinski A, Deorowicz S, Gudyś A. PHIST: fast and accurate prediction of prokaryotic hosts from metagenomic viral sequences. Bioinformatics. 2022;38(5):1447–1449. doi: 10.1093/bioinformatics/btab837 34904625 PMC8826084

[18] Lu C, Zhang Z, Cai Z, Zhu Z, Qiu Y, Wu A, et al. Prokaryotic virus host predictor: a Gaussian model for host prediction of prokaryotic viruses in metagenomics. BMC biology. 2021;19:1–11. doi: 10.1186/s12915-020-00938-6 33441133 PMC7807511

[19] Krizhevsky A, Sutskever I, Hinton GE. Imagenet classification with deep convolutional neural networks. Communications of the ACM. 2017;60(6):84–90. doi: 10.1145/3065386

[20] Ruohan W, Xianglilan Z, Jianping W, Shuai Cheng L. DeepHost: phage host prediction with convolutional neural network. Briefings in Bioinformatics. 2022;23(1):bbab385. doi: 10.1093/bib/bbab385 34553750

[21] Wang W, Ren J, Tang K, Dart E, Ignacio-Espinoza JC, Fuhrman JA, et al. A network-based integrated framework for predicting virus–prokaryote interactions. NAR genomics and bioinformatics. 2020;2(2):lqaa044. doi: 10.1093/nargab/lqaa044 32626849 PMC7324143

[22] Shang J, Sun Y. Predicting the hosts of prokaryotic viruses using GCN-based semi-supervised learning. BMC biology. 2021;19(1):1–15. doi: 10.1186/s12915-021-01180-4 34819064 PMC8611875

[23] Shang J, Sun Y. CHERRY: a Computational metHod for accuratE pRediction of virus-pRokarYotic interactions using a graph encoder-decoder model. arXiv preprint arXiv:220101018. 2022;. 10.1093/bib/bbac182 PMC9487644 35595715

[24] Zhou J, Cui G, Hu S, Zhang Z, Yang C, Liu Z, et al. Graph neural networks: A review of methods and applications. AI open. 2020;1:57–81. doi: 10.1016/j.aiopen.2021.01.001

[25] He X, Deng K, Wang X, Li Y, Zhang Y, Wang M. Lightgcn: Simplifying and powering graph convolution network for recommendation. In: Proceedings of the 43rd International ACM SIGIR conference on research and development in Information Retrieval; 2020. p. 639–648.

[26] JMLR Workshop and Conference Proceedings. Understanding the difficulty of training deep feedforward neural networks; 2010.

[27] Jaiswal A, Babu AR, Zadeh MZ, Banerjee D, Makedon F. A survey on contrastive self-supervised learning. Technologies. 2020;9(1):2. doi: 10.3390/technologies9010002

[28] Are graph augmentations necessary? simple graph contrastive learning for recommendation; 2022.

[29] Oord Avd, Li Y, Vinyals O. Representation learning with contrastive predictive coding. arXiv preprint arXiv:180703748. 2018;.

[30] Kingma DP, Ba J. Adam: A method for stochastic optimization. arXiv preprint arXiv:14126980. 2014;.

[31] Baker DN, Langmead B. Dashing: Fast and Accurate Genomic Distances with HyperLogLog. Cold Spring Harbor Laboratory. 2018;(1). 10.1186/s13059-019-1875-0 PMC6892282 31801633

[32] Duan Y, Llorente C, Lang S, Brandl K, Chu H, Jiang L, et al. Bacteriophage targeting of gut bacterium attenuates alcoholic liver disease. Nature. 2019;575(7783):505–511. doi: 10.1038/s41586-019-1742-x 31723265 PMC6872939

[33] Zheng DW, Dong X, Pan P, Chen KW, Fan JX, Cheng SX, et al. Phage-guided modulation of the gut microbiota of mouse models of colorectal cancer augments their responses to chemotherapy. Nature biomedical engineering. 2019;3(9):717–728. doi: 10.1038/s41551-019-0423-2 31332342

[34] Dong X, Pan P, Zheng DW, Bao P, Zeng X, Zhang XZ. Bioinorganic hybrid bacteriophage for modulation of intestinal microbiota to remodel tumor-immune microenvironment against colorectal cancer. Science Advances. 2020;6(20):eaba1590. doi: 10.1126/sciadv.aba1590 32440552 PMC7228756

[35] Shkoporov AN, Hill C. Bacteriophages of the human gut: the “known unknown” of the microbiome. Cell host & microbe. 2019;25(2):195–209. doi: 10.1016/j.chom.2019.01.017 30763534

[36] Shen J, Zhang J, Mo L, Li Y, Li Y, Li C, et al. Large-scale phage cultivation for commensal human gut bacteria. Cell Host & Microbe. 2023;31(4):665–677. doi: 10.1016/j.chom.2023.03.013 37054680

[37] Antipov D, Raiko M, Lapidus A, Pevzner PA. Metaviral SPAdes: assembly of viruses from metagenomic data. Bioinformatics. 2020;36(14):4126–4129. doi: 10.1093/bioinformatics/btaa490 32413137

[38] Auslander N, Gussow AB, Benler S, Wolf YI, Koonin EV. Seeker: alignment-free identification of bacteriophage genomes by deep learning. Nucleic acids research. 2020;48(21):e121–e121. doi: 10.1093/nar/gkaa856 33045744 PMC7708075

[39] Roux S, Enault F, Hurwitz BL, Sullivan MB. VirSorter: mining viral signal from microbial genomic data. PeerJ. 2015;3:e985. doi: 10.7717/peerj.985 26038737 PMC4451026

[40] Marbouty M, Thierry A, Millot GA, Koszul R. MetaHiC phage-bacteria infection network reveals active cycling phages of the healthy human gut. Elife. 2021;10:e60608. doi: 10.7554/eLife.60608 33634788 PMC7963479

[41] Schwarzer D, Buettner FF, Browning C, Nazarov S, Rabsch W, Bethe A, et al. A multivalent adsorption apparatus explains the broad host range of phage phi92: a comprehensive genomic and structural analysis. Journal of virology. 2012;86(19):10384–10398. doi: 10.1128/JVI.00801-12 22787233 PMC3457257

[42] Hoelzer K, Switt AIM, Wiedmann M, Boor KJ. Emerging needs and opportunities in foodborne disease detection and prevention: From tools to people. Food microbiology. 2018;75:65–71. doi: 10.1016/j.fm.2017.07.006 30056965

[43] Lee H, Ku HJ, Lee DH, Kim YT, Shin H, Ryu S, et al. Characterization and genomic study of the novel bacteriophage HY01 infecting both Escherichia coli O157: H7 and Shigella flexneri: potential as a biocontrol agent in food. PloS one. 2016;11(12):e0168985. doi: 10.1371/journal.pone.0168985 28036349 PMC5201272

[44] Yamaki S, Yamazaki K, Kawai Y. Broad host range bacteriophage, EscoHU1, infecting Escherichia coli O157: H7 and Salmonella enterica: Characterization, comparative genomics, and applications in food safety. International Journal of Food Microbiology. 2022;372:109680. doi: 10.1016/j.ijfoodmicro.2022.109680 35512432

[45] Zhang Y, Zou G, Islam MS, Liu K, Xue S, Song Z, et al. Combine thermal processing with polyvalent phage LPEK22 to prevent the Escherichia coli and Salmonella enterica contamination in Food. Food Research International. 2023; p. 112454. doi: 10.1016/j.foodres.2022.112454 36869473

